# Removal of Emulsified Oil from Water by Fruiting Bodies of Macro-Fungus (*Auricularia polytricha*)

**DOI:** 10.1371/journal.pone.0095162

**Published:** 2014-04-17

**Authors:** Xunan Yang, Mengting Guo, Yinghai Wu, Qunhe Wu, Renduo Zhang

**Affiliations:** 1 School of Environmental Science and Engineering, Guangdong Provincial Key Laboratory of Environmental Pollution Control and Remediation Technology, Sun Yat-sen University, Guangzhou, China; 2 South China Institute of Environmental Sciences, Ministry of Environmental Protection, Guangzhou, China; University of Kansas, United States of America

## Abstract

The aim of this study was to investigate the feasibility of utilizing the fruiting bodies of a jelly macro-fungus *Auricularia polytricha* as adsorbents to remove emulsified oil from water. The effects of several factors, including temperature, initial pH, agitation speed, and adsorbent dosage, were taken into account. Results showed that the optimized conditions for adsorption of *A. polytricha* were a temperature of 35°C, pH of 7.5, and agitation speed of 100 rpm. The adsorption kinetics were characterized by the pseudo-first order model, which showed the adsorption to be a fast physical process. The Langmuir-Freundlich isotherm described the adsorption very well and predicted the maximum adsorption capacity of 398 mg g^−1^, under optimized conditions. As illustrated by scanning electron micrographs, the oil particles were adsorbed onto the hairs covering the bottom surface and could be desorbed by normal temperature volatilization. The material could be used as an emulsified oil adsorbent at least three times, retaining more than 95% of the maximum adsorption capacity. The results demonstrated that the fruiting bodies of *A*. *polytricha* can be a useful adsorbent to remove emulsified oil from water.

## Introduction

The presence of emulsified oils in natural water and wastewater is a significant environmental concern. Oils can enter the food chain to threaten human health and cause problems in wastewater treatment plants [1]. Oily water is usually generated by industries that process petroleum, metals, food and textiles [2]. Several methods of oil removal have been developed, including methods involving adsorbents. The method can separate oil and water and recover oil easily [3]. Activated carbon and synthetic sorbents (such as polypropylene and polyurethane foam) have been widely used as traditional adsorbents for oil removal [4]. Natural adsorbents, such as processed plant materials [2], [5], mineral materials [4], and chitosan [6], have been examined for their oil adsorption capacity and for any role they could come to play in sustainable environmental technology [7].

Fungal biomass has been used in many types of separation technology [8]–[10]. The large quantities of chitosan, chitin, phosphate, and polysaccharides on fungal cell walls make fungi an ideal adsorbent [11], [12]. Two chitosan-abundant micro-fungi, *Mucor rouxii* and *Absidia coerulea*, have been used in emulsified oil adsorption [12]. Macro-fungi, such as *Ganoderma lucidum* [13], *Pycnoporus sanguineus* [14], edible Agaric fungal species [15], [16], and *Auricularia polytricha* as well as *Tremella fuciformis* [17], have been promised as adsorbents in the removal metals from aqueous solutions. However, little information is available regarding the adsorption of emulsified oil by macro-fungi. Although micro-fungi are easy to grow and have a short cultivation time, the large size and great physical strength of macro-fungi make them easy to use as a bioadsorbent. *A*. *polytricha* is a jelly fungus, a member of Basidiomycota. These fungi form gelatinous fruiting bodies. When exposed to water, the dried horny texture of the fungus becomes gelatinous because of the polysaccharides, which compose 60%–70% of the dry fruiting body [17]. The fruiting body of *A. polytricha* is pileate, and its upper surface can be smooth or wrinkled. Its lower surface is covered with short, bushy hairs, which may facilitate oil adsorption. Because it is an edible jelly fungus with high nutrient content, *A. polyticha* is cultivated on a large scale throughout the world. During commercial production, large amounts of fruiting body residue are generated. These may be suitable for use as adsorbents of emulsified oil.

The objective of this study was to investigate the feasibility of using the fruiting bodies of *A. polytricha* as adsorbents to remove emulsified oil from water. First, the effects of various experimental factors (i.e., adsorbent dosage, emulsified oil concentration, temperature, initial pH, and agitation speed) on adsorption performance of the fruiting bodies were exmined. Second, the adsorption kinetics and isotherm processes were evaluated. Finally, the desorption and reusability of the adsorbent were tested through multiple cycles of adsorption-desorption.

## Materials and Methods

### Experimental materials

Mineral oil was emulsified with oleic acid and triethanolamine using tap water as described by Biswas [18]. This type of emulsified oil had been used in biosorption studies [9], [12]. Dry fruiting bodies of *A. polytricha* (Agricultural Culture Collection of China, ACCC50140) were soaked in and then washed with tap water. Each soaked fruiting body was cut into pieces about 0.5 cm ×0.5 cm and air dried. Before the experiment, the fruiting body was soaked again. The structural and surface characteristics of the fruiting body were visualized using a scanning electron microscope (SEM, JSM-6330F, JEOL, Japan) at 15 kV. The materials were freeze-dried in the same way to avoid additional oil desorption for SEM preparation before and after adsorption, and after thermal volatilization.

### Biosorption studies

Biosorption experiments were conducted under different conditions, including combinations of different biomass dosages of *A. polytricha*, initial concentrations of emulsified oil, solution temperatures, agitation speeds, and initial pH values. Each biosorption experiment was conducted with a known amount of biomass in 50 mL oil-in-water emulsion for a set contact time. After biosorption, the oil-in-water emulsion was filtered through a 1.5 mm steel micro-filter. A control with no biomass was also set up for each run. The filtrate was extracted with tetrachloroethylene (ultra-resi analyzed) and then analyzed for oil concentration using an oil content analyzer (ASTAR IR200A, China, LOD  = 0.01 mg/L, RSD ≤2%). For each treatment, experiments were conducted in triplicate and the mean values were used for data analysis. The adsorption capacity of *A. polytricha* was determined as follows:
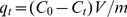
(1)Here, *q_t_* is the adsorption capacity (expressed as mg of oil per g of adsorbent) (mg g^−1^) within the contact time *t*, *C*_0_ is the initial concentration of emulsified oil (mg L^−1^), *C_t_* is the residual oil concentration in the solution after the adsorption experiment, *V* is the volume of the oil-in-water emulsion (L), and *m* is the dry weight of the adsorbent (g).

The Taguchi method with a L_16_ (4^5^) orthogonal array was employed to investigate the effect of different variables on biosorption of *A. polytricha* and an optimized condition of biosorption for kinetic and isotherm studies [19]. Five variables were evaluated at four levels each, i.e., dosages of the fungal biomass (0.2, 0.5, 0.7, and 1.0 g), initial oil concentrations (200, 500, 700, and 1000 mg L^−1^), initial pH values (5.5, 6.5, 7.5, and 8.5), temperatures (5, 15, 25, and 35°C), and speeds of a platform shaker (0, 50, 100, and 150 rpm). They are arranged in an orthogonal array in Table 1. Using the L_16_ (4^5^) orthogonal array design, four runs were set for each variable level. Biosorption experiments were conducted using the combined conditions given in Table 1 and a contact time of 1.5 h. The effectiveness statistics were used to measure the effect of the variable levels on the adsorption capacity. The effectiveness statistics of each variable were calculated as follows:
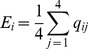
(2)Here, *E_i_* is the effectiveness of a variable at the *i*th level, *q_ij_* is the adsorption capacity of the *j*th run at the *i*th level measured at the end of the contact time. A higher effectiveness value indicated a higher adsorption capacity at the level of that variable. The range statistics were used to compare the effect among the variables on the adsorption capacity. The range statistics of each variable were calculated as the difference between the maximum and minimum *E_i_* values. A higher value of the range statistics indicated greater influence of the variable on adsorption.

**Table 1:** The L_16_(4^5^) orthogonal array for the experimental design.

ID	Temp.^a^ (°C)	Init. Conc. (mg L^−1^)	Dosage (g)	pH	Agita. Speed (rpm)	Adsorp. Capacity
						(mg g^−1^)
1	5	200	0.2	5.5	0	30.67
2	5	500	0.5	6.5	50	38.95
3	5	700	0.7	7.5	100	40.39
4	5	1000	1.0	8.5	150	41.38
5	15	200	0.5	7.5	150	14.81
6	15	500	0.2	8.5	100	100.1
7	15	700	1.0	5.5	50	22.55
8	15	1000	0.7	6.5	0	54.12
9	25	200	0.7	8.5	50	11.33
10	25	500	1.0	7.5	0	19.32
11	25	700	0.2	6.5	100	139.1
12	25	1000	0.5	5.5	150	82.78
13	35	200	1.0	6.5	100	8.41
14	35	500	0.7	5.5	150	25.67
15	35	700	0.5	8.5	0	55.66
16	35	1000	0.2	7.5	50	205.0

a Temp., Init. Conc., Agita. Speed, and Adsorp. Capacity denote the adsoption temperature, initial concentrations of emulsified oil, speed of agitation, and the adsoption capacity under the designed conditions, respectively.

The kinetic study was conducted under optimized conditions using a series of contact times: 0.5, 1, 2, 5, 10, 30, 60, and 90 s. The isotherm study was conducted under optimized conditions with different initial oil concentrations of 200, 700, 1000, 1200, 1500, 2000, 3000, and 3500 mg L^−1^.

To evaluate the stability of adsorption and desorption, saturated adsorbent (0.2 g adsorbed at the optimized condition) was transferred into 20 mL of water and 1 mol L^−1^ of H_2_SO_4_, NaOH, and NaCl aqueous solutions for 10 min, respectively, and desorption ratios were measured. To assess reusability, the following procedure was repeated several times: the used adsorbent was oven-dried at 45°C, soaked in water, and then used as adsorbent in emulsified oil again.

### Kinetic and isotherm models

Two kinetic models, the pseudo-first order model [20] and the pseudo-second order model [21], were used to characterize the adsorption kinetics of emulsified oil adsorbed by *A. polytricha*. The integrated forms of the two models were as follows:
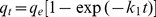
(3)

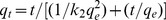
(4)Here, *q_e_* is the amount of emulsified oil adsorbed at the equilibrium (mg g^−1^), *k*_1_ (s^−1^) and *k*_2_ (g mg^−1^ s^−1^) are the rate constants of pseudo-first and pseudo-second order adsorptions, respectively.

Three commonly used isotherms, i.e., the Langmuir [22], Freundlich [23], and Langmuir-Freundlich equations [24], were used to describe the adsorption equilibrium:
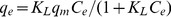
(5)

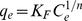
(6)

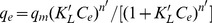
(7)Here *C_e_* is the residual emulsified oil concentration at the equilibrium (mg L^−1^), *q_m_* is the maximum adsorption capacity (mg g^−1^), *K_L_* is the Langmuir adsorption equilibrium constant (L mg^−1^), *K_F_* and *n* are the Freundlich constants representing the adsorption capacity (L^−1/n^ mg^− (1−1/n)^ g^−1^) and the adsorption intensity varying with the degree of heterogeneity, respectively, and *K_L_′* and *n′* are the Langmuir adsorption equilibrium constant (L mg^−1^) and the adsorption intensity in the Langmuir-Freundlich equation, respectively.

The coefficient of determination (*R^2^*) and the root mean square deviation (*RMSD*) were used to measure the ability of the models to fit the data accurately. The *RMSD* is calculated as follows:
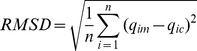
(8)Here, *n* is the number of experimental points, and *q_im_* and *q_ic_* are the *i*th measured and calculated values, respectively. A higher *R^2^* value and a lower *RMSD* value indicated a better fit result.

## Results and Discussion

### The effects of different factors on oil adsorption by *A. polytricha*

Effectiveness results based on the biosorption experiments using the combined conditions are shown in Table 2. According to the range statistics, the degree to which each of the factors influenced the adsorption of emulsified oil by *A. polytricha* was in the following order: biomass dosage > initial concentration of emulsified oil > temperature > agitation speed > initial pH. The adsorbent dosage is key to the adsorption process because a larger dosage brings more active sites but leads to more unsaturated adsorption [1], [25]. In this study, removals were similar even when dosage differed (77%–79%), but the adsorption capacity of *A. polytricha* decreased as dosage increased (Table 2).

**Table 2:** Results from the L_16_ (4^5^) orthogonal array experiments.

	Temperature (°C)	Init. Conc.^a^ (mg L^−1^)	Dosage (g)	pH	Agita. Speed (rpm)
					
*E*_1_ b	37.85	16.30	118.7	40.42	39.94
*E*_2_	47.90	46.01	48.05	60.14	69.45
*E*_3_	63.13	64.42	32.87	69.87	72.00
*E*_4_	73.68	95.82	22.92	52.12	41.16
Range	35.83	79.51	95.79	29.46	32.06

a Init. Conc. and Agita. Speed denote the initial concentrations of emulsified oil and the speed of agitation, respectively.

b *E_i_* (*i* = 1, 2, 3, 4) denotes the effectiveness (mg g^−1^) of the variables at the *i*th level.

Initial concentration can strongly affect the adsorption kinetics. At a high initial concentration, the gradient between the solution and adsorbent surface enhances emulsified oil diffusion through the film surrounding the adsorbent surface and in the spaces between the hairs [6]. Adsorption capacity was expected to increase with the initial oil concentrations reaching the equilibrium at a certain initial concentration [26]. Similar results were obtained in our experiments.

Temperature affects the adsorbent and adsorbate simultaneously. Rajaković-Ognjanović et al. suggested that the characteristics of organic sorbents become unstable at a high temperature and so decrease adsorption capacity [27]. However, at elevated temperatures, the interactions between adsorbent and adsorbate molecules become more intense thus increase the diffusion rate of the adsorbate molecules across the adsorbent surface [1]. In this study, the adsorption capacity of *A. polytricha* increased with the temperature (5°C to 35°C), indicating that the characteristics of *A. polytricha* were stable in this range of temperatures. The relatively high temperatures facilitated interaction between emulsified oil and the surface of *A. polytricha*. Ibrahim et al. observed similar results using an agricultural waste sorbent [1].

Mass transfer rates usually increase with agitation speed, but excessively vigorous agitation causes more adsorbate to be desorbed from the adsorbent surface [1]. Here, an agitation speed of 100 rpm was selected (Table 2). The initial pH is an important factor in the adsorption process. It works by influencing the surface properties of adsorbent (e.g., surface binding sites) and the stability of adsorbate simultaneously [7]. In this study, the effect of initial pH was less significant than that of the other selected factors and the initial pH values of 5.5, 6.5, 7.5, and 8.5 changed to 5.9, 6.7, 7.4, and 8.3, respectively, by the end of the contact period. Based on these results, an optimized set of conditions was established for the following experiments, including 0.2 g of biomass, 1000 mg L^−1^ of initial concentration, 35°C, 100 rpm of agitation speed, and initial pH of 7.5.

### Adsorption kinetics

**Figure 1:**
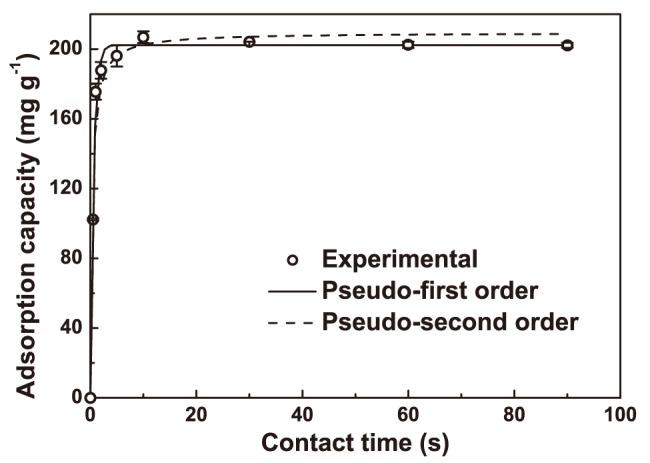
Kinetic models fitted with the data of adsorption conducted under the optimized conditions (i.e., 0.2 g of biomass, 1000 mg L^−1^ of initial oil concentration, 35°C, 100 rpm of agitation speed, and 7.5 of initial pH) in a period of 90 s. The bars represent the standard errors.

To study the equilibrium time of oil adsorption by *A. polytricha*, batch experiments were performed under the optimized conditions for periods of 90 s. As shown in Figure 1, the oil adsorption occurred at the primary rapid phase (about 2 s), followed by a relatively slow phase, and reached equilibrium within 10 s. The existence of bare surface of the adsorbent played a key role in this initially high rate of oil adsorption. However, adsorption sites became less available as the contact time increased, resulting in a slow adsorption phase [1]. During this slow adsorption phase, the breakage of oil droplets increased the interfacial area available for adsorption [28]. This represented the denaturation and reorganization of the bound oil at the interface to form spread films [29]. After 10 s, the surface adsorption sites of *A. polytricha* were saturated with oil particles and an equilibrium between adsorption and desorption was reached.

Two kinetic models were used to characterize the mechanism of adsorption reactions at the solid-solution interface in the adsorption of emulsified oil by *A. polytricha*. The *R* ^2^ and *RMSD* values in Table 3 indicated that the pseudo-first order model fit the data better than the pseudo-second order model, suggesting that the adsorption rate could be controlled by film diffusion [30]. The rate constant of sorption (*k*_1_ = 1.6 s^−1^) indicated fast physical adsorption.

**Table 3:** Fitting results of the kinetic and isotherm models to adsorption data of emulsified oil by *A. polytricha*.

Model	Parameter	Value
	Kinetic model	
Pseudo-first-order	*q_e_* (mg g^−1^)	202
	*k*_1_ (s^−1^)	1.62
	*R* ^2^	0.948
	*RMSD*	6.91
Pseudo-second-order	*q_e_* (mg g^−1^)	210
	*k*_2_ (g mg^−1^ s^−1^)	0.0137
	*R* ^2^	0.856
	*RMSD*	11.4
	Isotherm model	
Langmuir	*K_L_* (L mg^−1^)	0.00339
	*q_m_* (mg g^−1^)	487
	*R* ^2^	0.879
	*RMSD*	41.1
Freundlich	*K_F_* (L^−1/n^ mg^− (1−1/n)^ g^−1^)	26.4
	*N*	2.68
	*R* ^2^	0.720
	*RMSD*	62.6
Langmuir-Freundlich	*K_L_'* (L mg^−1^)	0.00493
	*n'*	2.39
	*q_m_* (mg g^−1^)	398
	*R* ^2^	0.985
	*RMSD*	13.4

### Equilibrium isotherm

**Figure 2:**
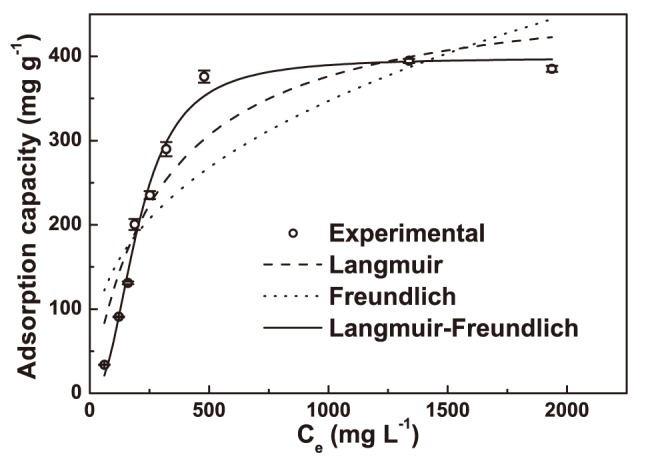
Isotherm models fitted with the data of adsorption of emulsified oil onto *A. polytricha* from a batch experiment conducted with different initial oil concentrations ranging from 200 to 3500 mg L^−1^ and other conditions: 0.2 g of biomass, 35°C, 100 rpm of agitation speed, and 7.5 of initial pH. The bars represent the standard errors.

To study the adsorption isotherm, experiments were conducted using different initial oil concentrations ranging from 200 to 3500 mg L^−1^ under other conditions: 0.2 g of biomass, 35°C, agitation speed of 100 rpm, and initial pH of 7.5 (Figure 2). Because the active sites were occupied gradually, oil adsorption increased as the initial concentrations increased, reaching the equilibrium at an initial concentration of about 2000 mg L^−1^. Based on the *R* ^2^ and *RMDS* values shown in Table 3, the Langmuir-Freundlich isotherm model fit the isotherm data best of any of the three models, indicating that a combined process of the Langmuir-type and Freundlich-type adsorption happens. The Langmuir-Freundlich model can predict a cooperative adsorption process involving to the adsorbate-adsorbate interactions [31]. The adsorbate-adsorbate interactions also occurred in this study because the adsorbed oil coalesced and formed droplets in the interspace of the microstructure on the surface of *A. polytricha* (see next section).

The maximum adsorption capacity predicted by the Langmuir-Freundlich model was 398 mg g^−1^ (Table 3). *A. polytricha* had a higher adsorption capacity than various inorganic materials (e.g., vermiculite and sepiolite), as reported in previous studies (Table 4). Despite a lower maximum adsorption capacity, *A. polytricha* was found to be much easier to handle than modified natural biomass. The cost of oil removal using *A. polytricha* was higher than using chitosan ($0.088 vs. $0.038 mg^−1^), but the market price of the former was much lower ($3.50 vs. $13.00 kg^−1^) [32]. To further support the use of *A. polytricha* over other materials in industrial scale, other factors, including the value of recovered oil, and costs of storage, operation, recycling and disposal, should be thoroughly investigated.

**Table 4:** Comparison of oil adsorption capacities of *A. polytricha* and some sorbents in the literature.

Adsorbent	Emulsified oil	Adsorption capacity (mg g^−1^)	Reference
Sepiolite	Motor oil	190	[5]
Expanded vermiculite	Mineral oil	163	[4]
Hydrophobized vermiculite	Mineral oil	108	[4]
Surfactant modified barley straw	Mineral oil	584	[2]
Chitosan powder	Palm oil mill effluent	3420	[6]
Chitosan flake	Palm oil mill effluent	1970	[6]
*A. polytricha*	Mineral oil	398	This study

The favorability of the adsorption can be characterized using the separation factor as follows [6]:
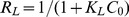
(9)Here, *C*_0_ is the initial concentration of emulsified oil, and *K_L_* is the Langmuir adsorption equilibrium constant (L mg^−1^). Because of the better fit provided by the Langmuir-Freundlich model, the *K_L_′* value shown in Table 3 was used to replace *K_L_* in Equation (9). The *R_L_* values calculated for different initial concentrations of emulsified oil are shown in Table 5. The results of 0<*R_L_*<1 indicated favorable adsorption of emulsified oil by the surface of *A. polytricha* [6].

**Table 5:** Results of the separation factor.

C_0_	R_L_
200	0.50
500	0.29
700	0.22
1000	0.17
1500	0.12
2000	0.09
3000	0.06
3500	0.05

### Oil leaching, desorption, and recycling

The desorption study showed the relative amounts of oil leaching in water and in solutions of 1 mol L^−1^ H_2_SO_4_, 1 mol L^−1^ NaOH, and 1 mol L^−1^ NaCl after 10 min exposure to be 0.61%, 0.62%, 1.4%, and 0.12%, respectively. The low amount of desorption of oil in water suggested that the oil adsorbed on the surface of *A. polytricha* had remained stable. The small relative amount of oil desorption in the acid, alkali, and salt solutions showed that the physical adsorption played the primary role in the adsorption of oil onto the surface of *A. polytricha*. For this reason, thermal volatilization desorption was used to desorb the oil from the surface. Oil spots were observed on the petri dish during oven drying.

**Figure 3:**
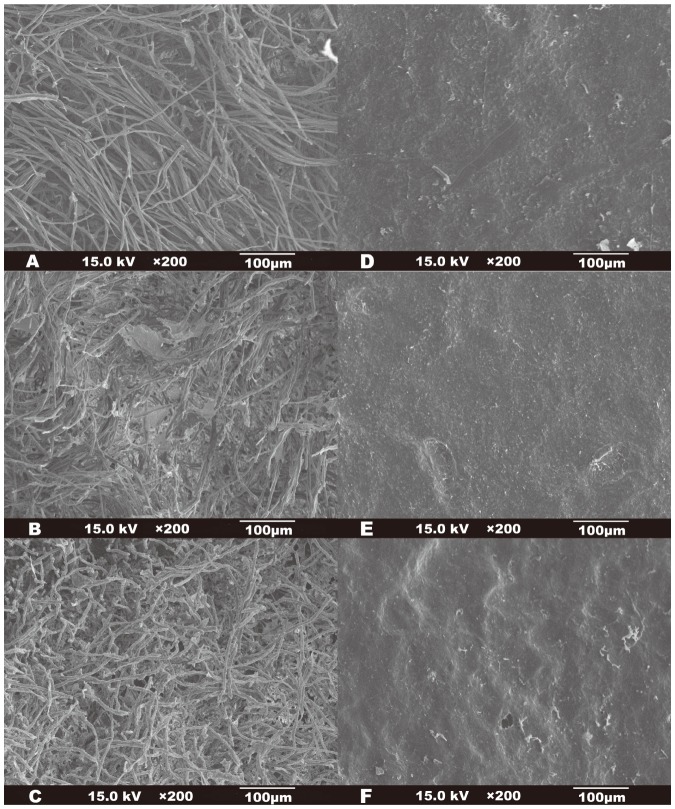
Scanning electron micrographs of *A. polytricha* lower surface (A) before oil adsorption, (B) after oil adsorption, and (C) after thermal volatilization desorption; and upper suface (D) before oil adsorption, (E) after oil adsorption, and (F) after thermal volatilization desorption.

To confirm the oil adsorption by *A. polytricha*, SEM micrographs were taken before and after adsorption and after thermal volatilization (Figure 3). The lower surface of *A. polytricha* is covered with hairs (Figure 3A), forming a 300 µm thick layer. The spaces between hairs were about 10–40 µm in size, which facilitated capillary action and molecule diffusion. After oil adsorption, most of the spaces were covered with muddy oil droplets (Figure 3B). The images given below show that oil was adsorbed by the lower surfaces of *A. polytricha* into the spaces between the hairs and developed into a layer of coalescent substance. After oven-drying, oil residue still remained on the hairs and in the spaces between the hairs, which had decreased in size (Figure 3C). There was no obvious change in the upper surface of *A. polytricha* during adsorption and desorption (Figure 3D–F).

Recycling of *A. polytricha* was tested through five adsorption (at 1000 mg L^−1^ initial oil concentration) and thermal volatilizing desorption cycles. The adsorption capacities of the first three cycles were 206, 199, and 195 mg L^−1^, respectively, and those of the fourth and fifth cycles decreased to 73% (150 mg L^−1^) and 57% (118 mg L^−1^) of the first cycle. These results suggested that the *A. polytricha* could be used as an emulsified oil adsorbent at least three times and retain more than 95% of its maximum adsorption capacity.

## Conclusion

According to the multi-factor effectiveness study, kinetic study, and scanning electron micrographs, the oil adsorption of *A. polytricha* fruiting body was a fast, film-diffusion-controlled physical process. The hairs covering the lower surface of *A. polytricha* played a key role in the oil adsorption. The oil particles were adsorbed onto the hairs and diffused into the spaces between, then coalesced into oil droplets. The adsorbed oil could be desorbed through normal temperature volatilization. The Langmuir-Freundlich isotherm predicted the maximum adsorption capacity of 398 mg g^−1^ under the optimized conditions and the adsorbate-adsorbate interactions occurred with favorable adsorption. The relatively high adsorption capacity, simple operation, large biomass, relatively low cost, and reusability suggested that the fruiting body of *A. polytricha* could be an ideal adsorbent for the removal of emulsified oil from contaminated water.
